# Effect of Catalytic Cylinders on Autothermal Reforming of Methane for Hydrogen Production in a Microchamber Reactor

**DOI:** 10.1155/2014/451919

**Published:** 2014-07-03

**Authors:** Yunfei Yan, Hongliang Guo, Li Zhang, Junchen Zhu, Zhongqing Yang, Qiang Tang, Xin Ji

**Affiliations:** ^1^Key Laboratory of Low-Grade Energy Utilization Technologies and Systems, Chongqing University, Ministry of Education, Chongqing 400030, China; ^2^College of Power Engineering, Chongqing University, Chongqing 400030, China

## Abstract

A new multicylinder microchamber reactor is designed on autothermal reforming of methane for hydrogen production, and its performance and thermal behavior, that is, based on the reaction mechanism, is numerically investigated by varying the cylinder radius, cylinder spacing, and cylinder layout. The results show that larger cylinder radius can promote reforming reaction; the mass fraction of methane decreased from 26% to 21% with cylinder radius from 0.25 mm to 0.75 mm; compact cylinder spacing corresponds to more catalytic surface and the time to steady state is decreased from 40 s to 20 s; alteration of staggered and aligned cylinder layout at constant inlet flow rates does not result in significant difference in reactor performance and it can be neglected. The results provide an indication and optimize performance of reactor; it achieves higher conversion compared with other reforming reactors.

## 1. Introduction

The development and application of micro-electro-mechanical system (MEMS) are collecting growing attentions. The reactor scale of order of millimeter offers a high degree of compactness and minimises heat as well as mass transport resistances [1], increasing effects of flame-wall interaction, and molecular diffusion are the major problems. Some scholars demonstrate the catalytic reforming of premixed hydrocarbon fuel and vapor to produce hydrogen. The addition of hydrogen maintains the stable and efficient hydrocarbon fuel combustion in microreactor.

Steam reforming of methane (SRM), an endothermic process, is well known as the main process for hydrogen production in industry [2]. While the partial oxidation of methane (POM) is an exothermic process, autothermal reforming of methane is the coupling for both. By combining two reactions, it is possible to operate under autothermal conditions, in which the enthalpy of SRM is balanced by that of POM. Extensive reviews about steam reforming of hydrocarbons discussed the conventional process; some scholars presented the multichannel reactors (MCR), typically dimensions which ranged from a few hundred micrometers to 3–5 mm, proved to be more efficiently to produce hydrogen [3–7]. A numerical method was also employed to simulate the catalytic partial oxidation of methane [8], exergy analysis was conducted to account for the heat recovery in waste steam. Ávila-Neto et al. [9] proposed a simulation code for the methane autothermal reforming where a methane conversion of about 50% can be reached by operating in the temperature range of 450–500°C. Murphy et al. [10] presented a ceramic microchannel reactor that combining heat-exchanger and catalytic-reactor functions to produce syngas; the research achieved high methane conversion. Yan et al. [11, 12] conducted numerical analysis of hydrogen-assisted catalytic combustion of methane; the effect of hydrogen addition on combustion of preheated mixtures of methane-hydrogen-air in a microcombustor was investigated including an elementary-step surface reaction mechanism. In previous works [13, 14], the activity of Ni-Al_2_O_3_ and Ni-MgO catalyst was tested for methane steam reforming using two different reaction systems and the highest reaction rates were found with monolith configuration. Besides, researches on several kinds of catalyst had also been carried out and the catalysts based on Ni, Rh, and Pt on various supports had been widely tested [15–17].

The above researches are mostly concerned with the chemical mechanism and theory of the reforming reaction. It demonstrated that the multicylinder affects the performance of reaction; in our work we simulate the reforming reaction in a microchamber reactor and carry out parametric studies that can provide guidance for practical application and similar reactor design.

## 2. Numerical Simulation 

### 2.1. Description of Reacting System

The reaction system considered in this work is the endothermic steam reforming of methane and methane catalytic combustion taking place in a microchamber with multicylinder inside. The main chemical reactions involved in the process are

 methane steam reforming:
  (1) $$\begin{array}{c} {\text{CH}}_{4}+{\text{H}}_{2}\text{O}⟷\text{CO}+3{\text{H}}_{2},\;\;\Delta H=+206.1\;\text{kJ}/\text{mol}, \end{array}$$
 methane partial oxidation:
  (2) $$\begin{array}{c} {\text{CH}}_{4}+0.5{\text{O}}_{2}⟷\text{CO}+2{\text{H}}_{2},\;\;\Delta H=-35.2\;\text{kJ}/\text{mol}, \end{array}$$
 methane autothermal reforming:
  (3) $$\begin{array}{c} {\text{CH}}_{4}+0.5{\text{O}}_{2}⟷\text{CO}+2{\text{H}}_{2},\;\;\Delta H=-35.2\;\text{kJ}/\text{mol}, \end{array}$$
  (4) $$\begin{array}{c} {\text{CH}}_{4}+{\text{H}}_{2}\text{O}⟷\text{CO}+3{\text{H}}_{2},\;\;\Delta H=+206.1\;\text{kJ}/\text{mol}. \end{array}$$

**Figure 1:**
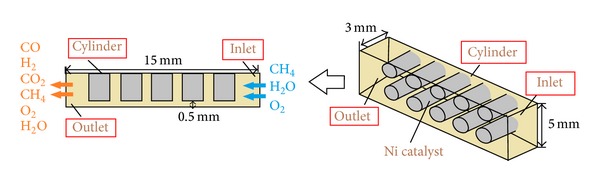
The schematic drawing of the microchamber reactor.

In this study, steam reforming of methane and partial oxidation of methane are coupled and the overall process is endothermic.  Figure 1 presents schematically the structure of the microchamber reactor: the mixed gas flow into the microchamber (length 15 mm, width 5 mm, and height about 3 mm) at inlet; two rows and five columns of cylinders (height 2.5 mm, radius and spacing various from 0.25 mm ~ 0.75 mm and 0.7 mm ~ 1.1 mm), covered with Ni-based catalyst, are placed to adjust the performance and thermal behavior of reactor.

The mechanism of reforming reaction with methane, steam, and oxygen over Ni catalyst dominates the reaction in microchamber reactor. The inner flow field is calculated according to following fundamental assumptions: steady state is considered for reactor operation; fully developed laminar flow is employed in microchamber reactor; the flow is incompressible and the gravitational influence is neglected; the walls within the chamber are catalytic surface and in constant temperature; the effect of volume force and dissipation function is neglected; no phase change occurred from gas to liquid phases; the basic operating parameters used in this paper are given in Table 1.

In the present work, analysis on methane autothermal reforming has been carried out to assess the performance of the microchamber reactor by varying the structural variables. In particular, results including methane conversion and mass fraction of methane/hydrogen have been studied.

### 2.2. Mesh Generation

A three-dimensional model of microchamber reactor with 1 : 1 proportions is built using the CFD preprocessing software GAMBIT, which is used for the three-dimensional flow passage and grid generation. FLUENT is based on the finite volume method and used to conduct the full passage numerical simulations.

**Figure 2:**
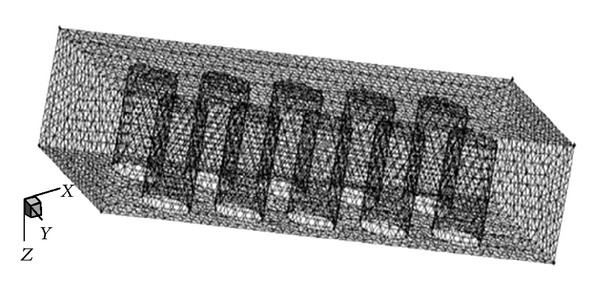
Three-dimensional mesh of the microchamber reactor.

The solution domain is divided into limited control volumes by grids. Thus grid generation has a great influence on the calculation accuracy and stability. A grid-independent study confirmed that grids provided sufficient grid independency. The grid independence is examined with 0.3, 0.4, 0.5, and 0.6 mm of interval size, respectively. As given in Table 2, the accuracy of the calculation is confirmed as the difference of methane conversion is 0.8% with interval size of 0.3 mm and 0.6 mm; thus, the interval size of 0.3 mm is adopted. To improve the computing accuracy, the mesh consisting of 48638 hybrid forms of triangular and hexahedral elements is adopted with special care for meshing around cylinders, as shown in Figure 2. In addition, grid point distributions near the catalytic surface (surface of cylinders and walls) are fined for accuracy.

The simulation carried out in the Fluent 6.3 environment and is integrated with Chemkin programs including mechanism of multiple reactions of methane, steam, and oxygen on Ni catalyst.

### 2.3. Governing Equations

To describe the reaction process of the methane, water vapor and the oxygen, the governing equations are given by

 continuous equation:
  (5) $$\begin{array}{c} \frac{\partial \rho }{\partial t}+\frac{\partial }{\partial x_{i}}\left(\rho u_{i}\right)=0 \end{array}$$
 component equation:
  (6) $$\begin{array}{c} \frac{\partial }{\partial t}\left(\rho Y_{i}\right)+\nabla \left(\rho \vec{u}Y_{i}\right)=-\nabla \vec{J_{i}}+R_{i} \end{array}$$
 momentum equation:
  (7) $$\begin{array}{c} \frac{d\left(\rho \vec{V}\right)}{dt}=-\text{grad}\;p+\nabla^{2}\left(\mu \vec{V}\right) \end{array}$$
 energy equation:
  (8) $$\begin{array}{c} \rho \frac{Dh}{Dt}-\frac{\partial p}{\partial t}=\frac{\partial }{\partial x_{i}}\left(\lambda \frac{\partial T}{\partial x_{j}}\right)+\frac{\partial }{\partial x_{j}}\left(\sum_{i}D\rho \frac{\partial Y_{i}}{\partial x_{j}}h_{i}\right)+q \end{array}$$
 gas state equation:
  (9) $$\begin{array}{c} p=\rho RT\sum \frac{Y_{i}}{M_{i}} \end{array}$$

In (7), (8), and (9), the variable *p* is pressure; *T* is temperature; *ρ* is the density; *q* in (8) is the heat; *h* is the enthalpy; *λ* is the thermal conductivity; *u* is the flow rate; *μ* in (7) is the dynamic viscosity; *R* in (6), (9) is the gas constant; *Y*_*i*_ is the mass fraction of the component *i* and ∑*Y*_*i*_ = 1; *J*_*i*_ in (6) is the diffusion flux of component *i*, which is caused by the concentration gradient and can be calculated by

(10) $$\begin{array}{c} J_{i}=-\rho D_{i}\nabla Y_{i}, \end{array}$$

where the variable *D*_*i*_ is the diffusion coefficient of the component *i* in the mixture.

Arrhenius equation is used to calculate chemical source term for laminar finite rate model, *R*_*i*_ in (11) is the net production rate of reaction *i*, which can be calculated by a sum of Arrhenius reaction source term in *N*_*r*_ chemical reactions

(11) $$\begin{array}{c} R_{i}=M_{i}\overset{N_{r}}{\underset{i=1}{\sum }}{\overset{\wedge }{R}}_{i,r}, \end{array}$$

where *M*_*i*_ is the molecular weight of the material *i* and ${\overset{\wedge }{R}}_{i,r}$ in (11) is the generation or decomposition rate of material *i* in the formula *r* and is given by

(12) $$\begin{array}{c} {\overset{\wedge }{R}}_{i,r}=\Gamma \left(v_{i,r}^{\prime }-v_{i,r}^{\prime \prime }\right)\left\{k_{f,r}\overset{N_{r}}{\underset{j=1}{\prod }}\left[C_{j,r}\right]\eta_{j,r}^{\prime }-k_{b,r}\overset{N_{r}}{\underset{j=1}{\prod }}\left[C_{j,r}\right]\eta_{j,r}^{\prime \prime }\right\}, \end{array}$$

where Γ is the net effect of the third substance on the reaction rate; *v*_*i*,*r*_′ is the stoichiometric coefficient of reactant *i* in reaction *r*; *v*_*i*,*r*_′′ is the stoichiometric coefficient of resultant *i* in reaction *r*; *k*_*f*,*r*_ is the forward reaction rate in reaction *r*; *k*_*b*,*r*_ is the backward reaction rate in reaction *r*; *η*_*j*,*r*_′ is the forward reaction speed index of each reactant or product *j* in reaction *r*; *η*_*j*,*r*_′′ is the backward reaction speed index of each reactant or product *j* in reaction *r*; *C*_*j*,*r*_ is the molar concentration of each reactant or product *j* in reaction *r*.

## 3. Results and Discussion

### 3.1. Effect of Cylinder Radius

It identified that the location of the catalytic cylinders placed in microchamber may offer a degree of flexibility to adjust the temperature profile and prevent the detrimental reverse reaction in the endothermic side, avoiding at the same time severe hot spots [18]. The reactor performance with cylinder radius of 0.25, 0.50, and 0.75 mm are explored and the operating parameters are shown in Table 3.

**Figure 3:**
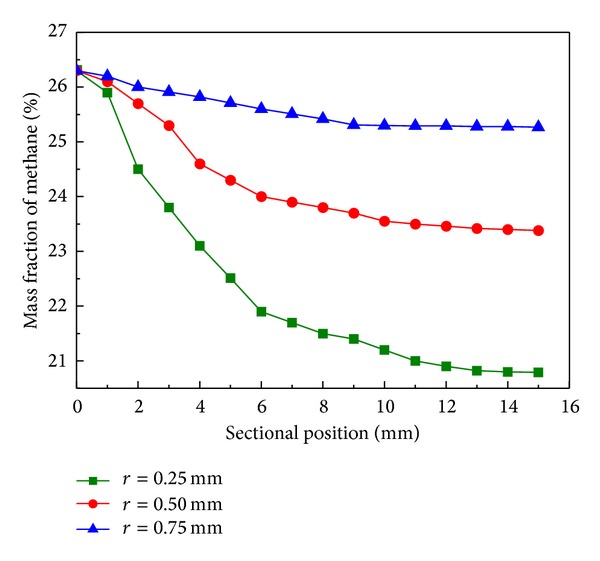
Mass fraction of methane along sectional position with cylinder radius of 0.25 mm (■), 0.50 mm (●), and 0.75 mm (▲) at reaction time of 3 s.

**Figure 4:**
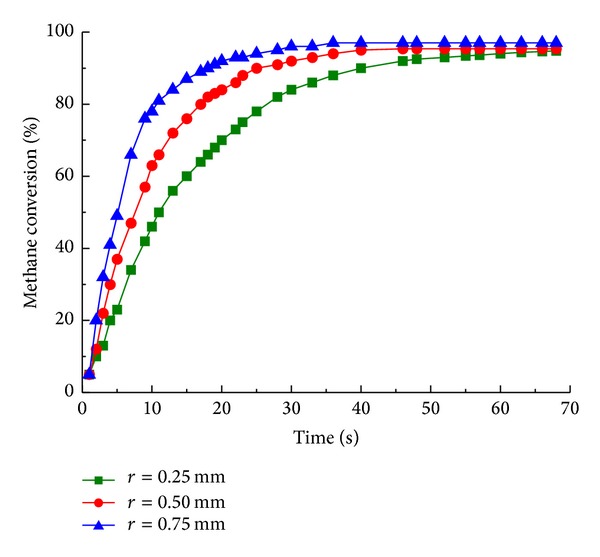
Methane conversion with cylinder radius of 0.25 mm (■), 0.50 mm (●), and 0.75 mm (▲) for different reaction time.

The numerical analysis has been carried out in order to verify the effect of cylinder radius (0.25 mm, 0.50 mm, and 0.75 mm) on reforming reaction. As shown in Figure 3, the mass fraction of methane decreases from 26% to 21% with cylinder radius of 0.75 mm at 3 s, while cylinder radius of 0.50 mm and 0.25 mm decreases to 23.5% and 25.3%, indicating that larger cylinder radius (from 0.25 mm to 0.75 mm) promotes the reactor performance. The methane conversion with different cylinder radius generally achieves 97% (see Figure 4) with operating temperature of 1190 K, higher than the conventional research [8, 19], partially due to the adjusted temperature profile and reaction heat flux caused by catalytic cylinders. Larger cylinder radius corresponds to more catalytic surface and the time to steady state reaction is decreased from 50 s to 30 s. Hence, expanding the cylinder radius is one option to improve reforming performance, subject to limited physical size of micro-chamber reactor.

**Figure 5:**
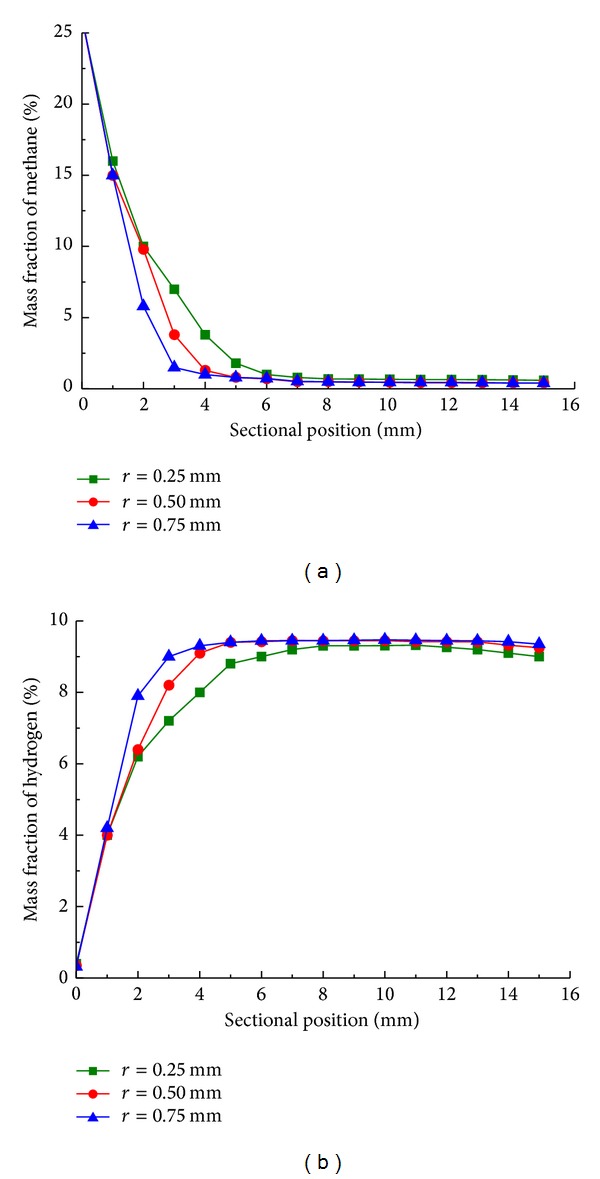
Mass fraction of (a) methane and (b) hydrogen along sectional position with cylinder radius of 0.25 mm (■), 0.50 mm (●), and 0.75 mm (▲) in steady state.

As shown in Figures 5(a) and 5(b), it can be seen that larger cylinder radius typically increased hydrogen yield. Particularly at sectional position of 3 mm, the mass fraction of hydrogen with cylinder radius of 0.75 mm is 2% higher than that of 0.25 mm, the mass fraction of methane with cylinder radius of 0.25 mm is 7% higher than that of 0.75 mm, indicating that larger cylinder radius results in increase of conversion mainly due to more efficient heat transfer. Rather small differences in mass fraction of methane and hydrogen are observed after 8 mm in steady state. The methane conversion and mass fraction of hydrogen with different radius are reported in Table 4, the effect of cylinder radius can be neglected in steady state. Thus the alteration of the cylinder radius does not affect significantly the outlet conversion and outlet reactor performance, partially for that reactant molecules have enough time to reach the wall before they exit the reactor [18].

### 3.2. Effect of Cylinder Spacing

The study has been carried out in order to verify the effect of cylinder spacing. The cylinder spacing is concerned with catalyst loading and flow field distribution in microchamber, turbulent flow and hot spots occurs when not properly designed [18]. The spacing of catalytic cylinders are 0.7, 0.9, and 1.1 mm, respectively, all other parameters are kept in constant and presented in Table 5.

**Figure 6:**
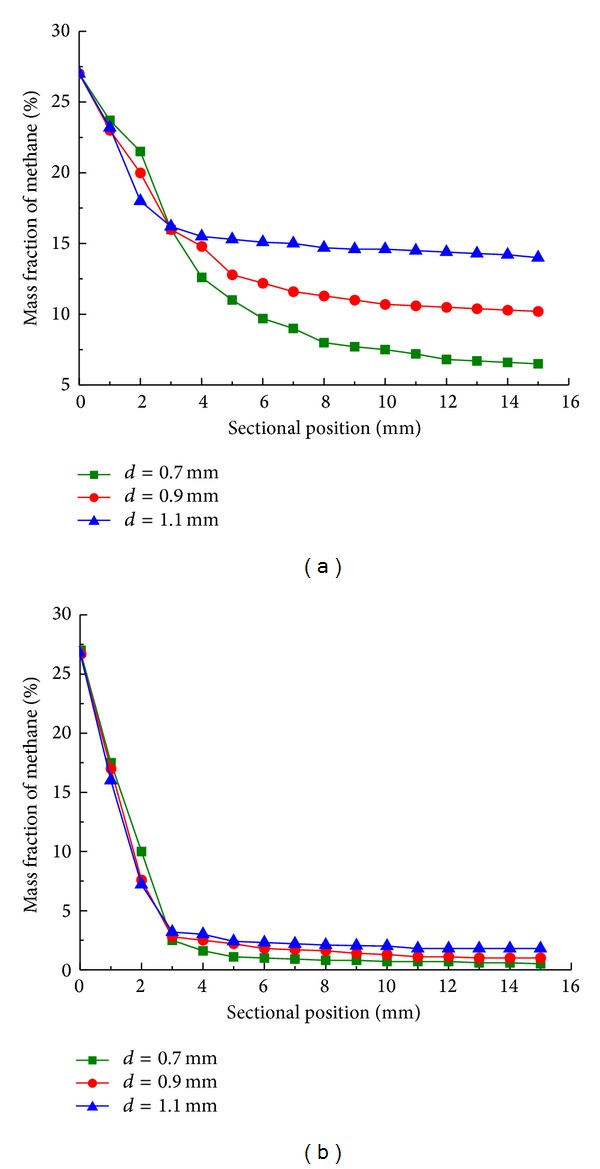
Mass fraction of methane along sectional position with cylinder spacing of 0.7 mm (■), 0.9 mm (●), and 1.1 mm (▲) at reaction time of 5 s (a) and 25 s (b).

Figures 6(a) and 6(b) show results of a series of numerical simulations. Mass fraction of methane with cylinder spacing of 0.7, 0.9, and 1.1 mm are plotted on sectional position along reactor length at reaction time of 5 s and 25 s. Mass fraction of methane generally decreases from 26% to 16% (see Figure 6(a)) and coincides at 3 mm (reaction time of 5 s). After that methane mass fraction with cylinder spacing of 0.7 mm decreases from 16% to 7%, while the spacing of 0.9 mm and 1.1 mm moves downstream to 14% and 10%, this indicates that a decrease in cylinder spacing (from 1.1 mm to 0.7 mm) promotes the reactor performance at outlet section. As a contrast, the same alteration of cylinder spacing is made and mass fraction of methane is approaching at reaction time of 25 s (see Figure 6(b)), partially due to fully developed and adjusted flow field and temperature profile, thus rather small differences are observed after 3 mm.

**Figure 7:**
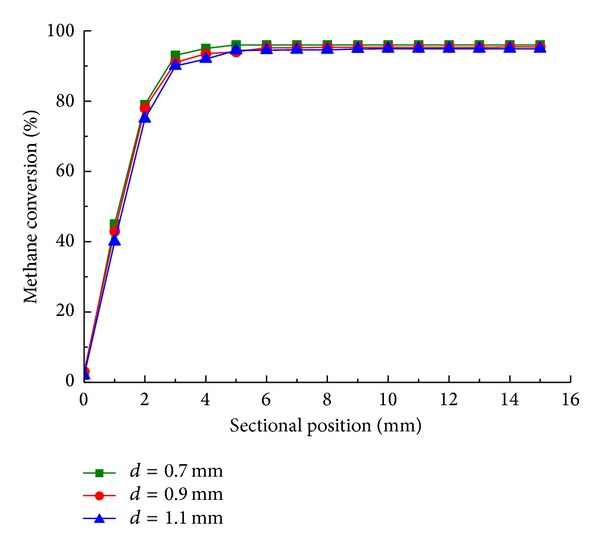
Methane conversion along sectional position with cylinder spacing of 0.7 mm (■), 0.9 mm (●), and 1.1 mm (▲) in steady state.

**Figure 8:**
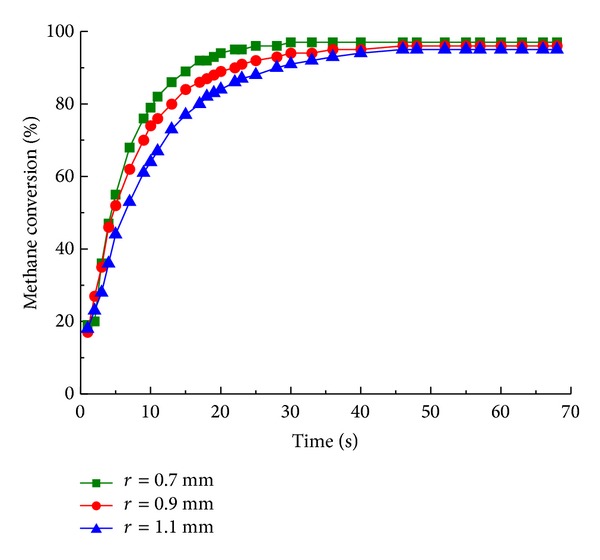
Methane conversion with cylinder spacing of 0.7 mm (■), 0.9 mm (●), and 1.1 mm (▲) for different reaction time.

As shown in Figure 7, alteration of cylinder spacing leads to no significant difference in steady state. It illustrates that the spacing of cylinders has no significant effect on the final state of reaction. The present work achieves a methane conversion of 95% (see Figure 7); it generally higher than improved performance of 91% and 93% conversion in previous experimental research [20, 21] (inlet flow rate 0.75 and 2; temperature of feed 873 K and 943 K), partially due to higher operating temperature. As shown in Figure 8, larger cylinder spacing results in higher conversion and the time to steady state is decreased (from 40 s to 20 s). The cylinder spacing is concerned with catalyst loading and more reactants contact catalytic surface with compact cylinder spacing. The methane conversion of cylinder spacing varied in Figure 8 approaches in steady state, indicating that the parameter is unlikely to optimize performance for the steady state reaction. The result also shows that operating temperature is the major factor limiting reforming performance; thus, a cooler reactor leads to lower final conversions.

### 3.3. Effect of Cylinder Layout

The arrangement of cylinders is separated with aligned and staggered layout. The reactor behavior and performance are studied for different cylinder layout and all other parameters shown in Table 6 are kept at constant value.

**Figure 9:**
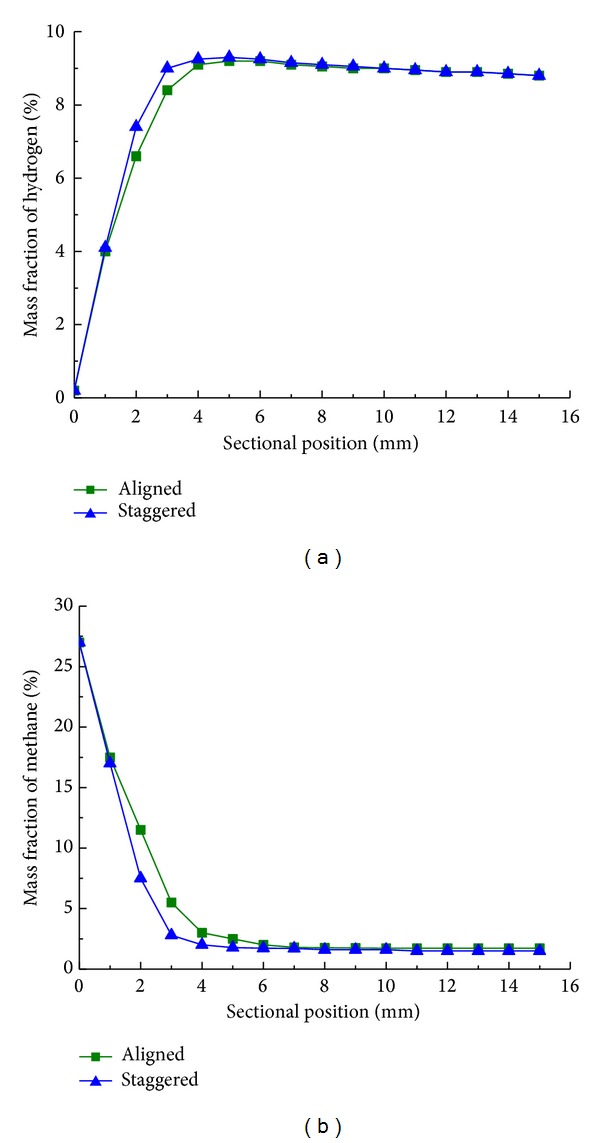
Mass fraction of (a) hydrogen and (b) methane along sectional position with aligned (■) and staggered (▲) layout of cylinder: reaction time *t*_*r*_ = 25 s.

Mass fraction of hydrogen and methane along sectional position with aligned and staggered layout at reaction time of 25 s is illustrated in Figures 9(a) and 9(b). It indicates a small benefit of staggered layout, since the mass fraction of hydrogen is generally higher in staggered arrangement before 6 mm. As a contrast, the mass fraction of methane is higher in aligned arrangement and after that no significant differences are observed. Methane partial oxidation reaction, an exothermic process, dominates the reaction initially and the staggered arrangement enhances heat transfer and the turbulence intensity.

**Figure 10:**
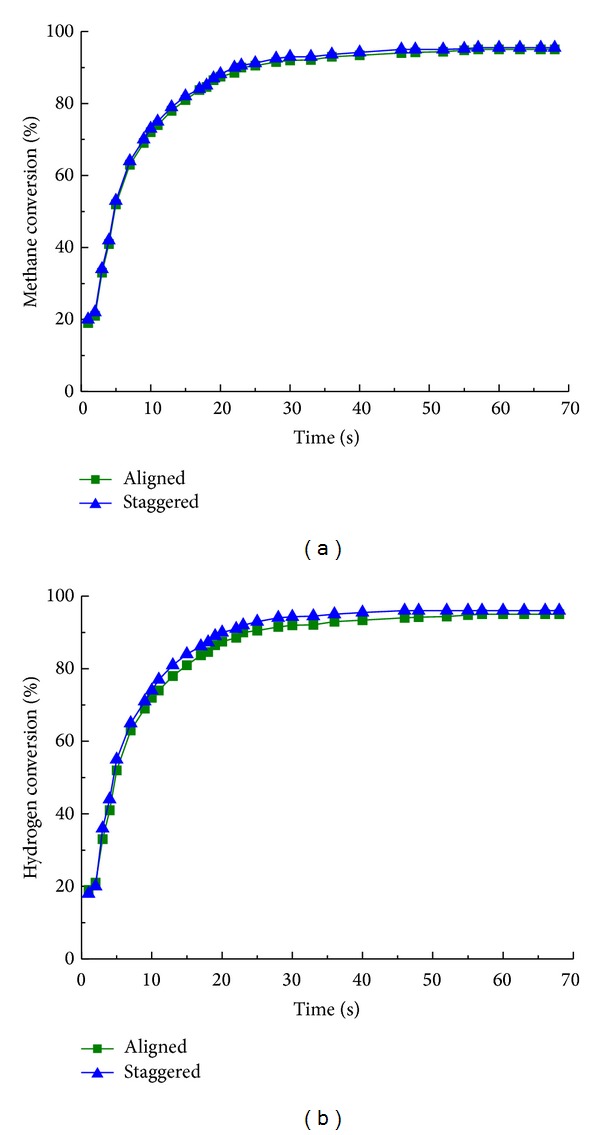
(a) Methane and (b) hydrogen conversion with aligned (■) and staggered (▲) layout of cylinder for different reaction time.

Methane and hydrogen conversion with staggered and aligned cylinder layout are illustrated in Figures 10(a) and 10(b); rather small differences with staggered and aligned cylinder layout are observed, indicating that the influence of cylinder layout for the overall reaction is negligible, despite the fact that the choice of aligned arrangement can reduce wear and tear on cylinders.

As shown in Table 7, the finalized structural parameters of cylinders in microchamber derived with cylinder radius of 0.75 mm, cylinder spacing of 0.7 mm, and aligned layout. The optimized parameters of the microchamber reactor provide guidance for its application and similar reactor design.

## 4. Conclusions

The performance of the reforming reaction was investigated by varying the cylinders covered with Ni catalysts in microchamber. It was concluded that larger cylinder radius resulted in more catalytic surface area and therefore it reduced the mass fraction of methane from 26% to 21%, while such effect was neglected for steady state reaction. The methane conversion with different cylinder radius generally achieves 97%. Smaller cylinder spacing enhanced the turbulence intensity and promoted the efficiency of heat transfer. Thus the reaction was fully developed and the time required to reach the steady state was decreased from 40 s to 20 s. At constant inlet flow rates, alteration of cylinder layout for staggered and aligned did not introduce significant differences in reactor performance. Staggered layout could partially enhance the methane conversion and hydrogen yield. While the promotion was insignificant after 6 mm and thus the effect of cylinder layout was negligible.

The results indicate that autothermal reforming of methane in microchamber was affected by catalytic cylinders inside and it should be properly designed; the optimized microchamber reactor with cylinder radius of 0.75 mm, cylinder spacing of 0.7 mm, and aligned layout was derived.

## Figures and Tables

**Table 1:** The basic operating parameters.

Inlet flow rate of mixed gas/(m*·*s^−1^)	Inlet temperature of mixed gas/(K)	Temperature of catalytic surface/(K)	Mole ratio (H : C : O)
0.005	300	1190	2 : 1 : 0.4

**Table 2:** Grid division in microchamber and methane conversion.

Interval size/(mm)	0.3	0.4	0.5	0.6
Methane conversion	99.57%	99.29%	99.14%	98.74%

**Table 3:** Operating parameters with different cylinder radius.

Number	Radius/(mm)	Inlet flow rate/(m*·*s^−1^)	Mole ratio (H : C : O)	Wall temperature/(K)	Cylinder spacing/(mm)	Cylinder layout
1	0.25	0.005	2 : 1 : 0.4	1190	0.9	Aligned
2	0.50	0.005	2 : 1 : 0.4	1190	0.9	Aligned
3	0.75	0.005	2 : 1 : 0.4	1190	0.9	Aligned

**Table 4:** Mass fraction of hydrogen and methane conversion with different cylinder radius.

Radius/(mm)	0.25	0.5	0.75
Methane conversion	99.37%	99.49%	99.74%
Mass fraction of hydrogen	9.26%	9.38%	9.33%

**Table 5:** Operating parameters with different cylinder spacing.

Number	Cylinder spacing/(mm)	Inlet flow rate/(m*·*s^−1^)	Mole ratio (H : C : O)	Wall temperature/(K)	Radius/(mm)	Cylinder layout
1	0.7	0.005	2 : 1 : 0.4	1190	0.75	Aligned
2	0.9	0.005	2 : 1 : 0.4	1190	0.75	Aligned
3	1.1	0.005	2 : 1 : 0.4	1190	0.75	Aligned

**Table 6:** Operating parameters with different cylinder layout.

Number	Cylinder layout	Inlet flow rate/(m*·*s^−1^)	Mole ratio (H : C : O)	Wall temperature/(K)	Radius/(mm)	Cylinder spacing/(mm)
1	Aligned	0.005	2 : 1 : 0.4	1190	0.75	0.7
2	Staggered	0.005	2 : 1 : 0.4	1190	0.75	0.7

**Table 7:** The finalized structure of microchamber.

Cylinder radius/(mm)	Cylinder spacing/(mm)	Cylinder layout
0.75	0.7	Aligned

## Nomenclature

### Variables

*C*_*j*,*r*_: The molar concentration of each reactant or product *j* in reaction *r* (−)
*D*_*i*_: Diffusion coefficient of component *I* (m^2^ s^−1^)
*h*: Enthalpy (kJ kg^−1^)
*J*: Diffusion flux (mol m^−2^ s^−1^)
*k*_*f*,*r*_: Forward reaction rate in reaction *r* (−)
*k*_*b*,*r*_: Backward reaction rate in reaction *r* (−)
*M*: Relative molecular mass (−)
*P*: Pressure (Pa)
*q*: Heat (kJ)
*R*: Universal gas constant = 8.314 (J mol^-1 ^K^−1^)
*R*_*i*_: Net production rate of reaction *i* (mol m^−3^ s^−1^)
*t*: Time (s)
*T*: Temperature (K)
*u*: Flowrate (m s^−1^)
*v*_*i*,*r*_′: The stoichiometric coefficient of reactant *i* in reaction *r* (−)
*V*_*i*,*r*_′′: The stoichiometric coefficient of resultant *i* in reaction *r* (−)
*Y*: Mass fraction (%).

### Greek Letters

*η*: Efficiency (%)
*η*_*i*,*r*_′: The forward reaction speed index of each reactant or product *j* in reaction *r* (−)
*η*_*i*,*r*_′′: The backward reaction speed index of each reactant or product *j* in reaction *r* (−)
*ρ*: Density (kg m^−3^)
∇: Gradient operator (−)
*λ*: Thermal conductivity (W m^−1^ k^−1^)
*μ*: Dynamic viscosity (N s m^−2^)
Γ: Index of the third substance on the reaction rate (−).
